# A biomechanical comparison of 360° stabilizations for corpectomy and total spondylectomy: a cadaveric study in the thoracolumbar spine

**DOI:** 10.1186/s13018-015-0240-6

**Published:** 2015-07-01

**Authors:** Jung-Hoon Kim, John M. Rhee, Yoshio Enyo, William C. Hutton, Sung-Soo Kim

**Affiliations:** Department of Orthopaedic Surgery, Ilsan Paik Hospital, Inje University, Goyang-si, Korea; Department of Orthopaedic Surgery, Emory Spine Center, Emory University, Atlanta, GA USA; Veterans Affairs Medical Center, Atlanta, GA USA; Department of Orthopaedic Surgery, Haeundae Paik Hospital, Inje University, Busan, Korea

**Keywords:** Corpectomy, Total spondylectomy, Biomechanics, Thoracolumbar spine, Stiffness, Stability

## Abstract

**Background:**

To date, there has been no adequate biomechanical model that would allow a quantitative comparison in terms of stability/stiffness between a corpectomy with the posterior column preserved and a total spondylectomy with the posterior column sacrificed. The objective of this study was to perform a biomechanical comparison of 360° stabilizations for corpectomy and total spondylectomy, using the human thoracolumbar spine.

**Methods:**

Five human cadaveric thoracolumbar spines (T8-L2) were tested according to the following loading protocol: axial compression, flexion, extension, lateral bending to the right and left, and axial rotation to the right and left. This loading protocol was applied three times. Each specimen was tested intact, after corpectomy, and after total spondylectomy. The relative stiffness of each motion segment was determined for each test.

**Results:**

There was no significant difference in stiffness after reconstruction of total spondylectomy versus corpectomy in our thoracolumbar model. Our construct consisted of an anterior cage and four-level pedicle screw instrumentation (two above and two below) and provided similar stiffness in both models. Despite the additional bone resection in a total spondylectomy versus corpectomy, the constructs did not differ biomechanically. Additionally, there was no significant difference in stiffness between the intact specimen and either reconstruction model.

**Conclusions:**

A classic corpectomy, which leaves the posterior column intact, is no better in terms of stability/stiffness than a total spondylectomy carried out using a shorter cage, followed by compression using posterior instrumentation.

## Background

Spine surgeons have used combined anterior and posterior surgery for the treatment of patients with spinal fractures, tumors, and deformities. Corpectomy is generally followed by anterior reconstruction with a strut graft or a cage and posterior instrumentation with pedicle screws. This procedure is performed to provide sufficient mechanical stability. However, total spondylectomy (complete resection of the affected vertebra, including the posterior column) by means of a posterior approach is sometimes preferred, because pedicle screws and a posterior approach technique can provide secure fixation. Posterior total spondylectomy can be useful in cases where the anterior approach is considered difficult, such as in cases of severe adhesions due to previous anterior surgery, suboptimal pulmonary function, severe kyphosis, and trunk shortening [1–3].

Classic corpectomy leaves the posterior column intact, thus, offering good biomechanical stability, since it provides support at both the front and back [4–6]. On the other hand, total spondylectomy sacrifices the posterior column and leads to complete loss of spinal continuity and stability during the operation. However, there is a report on total spondylectomy using a 10-mm shorter cage, followed by compression using posterior instrumentation; this resulted in spinal shortening and stability [7]. Ten millimeters of shortening is within the acceptable range for the spinal cord [8–10]. Despite the effectiveness of shortening, spine surgeons may question whether total spondylectomy with posterior instrumentation can provide stability comparable to combined anterior corpectomy and posterior instrumentation.

To date, there has been no adequate biomechanical model that would allow a quantitative comparison in terms of stability/stiffness between a corpectomy and a total spondylectomy. We decided to compare biomechanical stabilities using a thoracolumbar reconstructed spine (T9-L1), with anterior cage and multilevel posterior instrumentation after corpectomy (T11), and the same spine with a 10-mm shorter anterior cage and multilevel posterior instrumentation after total spondylectomy (T11).

## Materials and methods

### Cadaveric specimen

Thoracolumbar spines (T8-L2) were harvested from five fresh human cadavers (mean age, 82 years; range, 70–95 years; two females, three males). The specimens were examined grossly and radiographically to rule out malignancy or fractures that could have interfered with the results. The specimens were then frozen at −20 °C until the day before testing. After thawing to room temperature overnight, the surrounding soft tissues and muscles were dissected meticulously to preserve the osseous and ligamentous structures. The specimens were kept moist throughout the procedure.

### Bone mineral density

The bone mineral density (BMD) of all specimens was measured in a water bath, using dual-energy X-ray absorptiometry (GE Lunar Prodigy, GE Medical Systems, Madison, WI, USA). Using the World Health Organization definition of osteoporosis as BMD <0.8 g/cm^2^, all of the vertebrae were classified as osteoporotic.

### Biomechanical testing

**Fig. 1:**
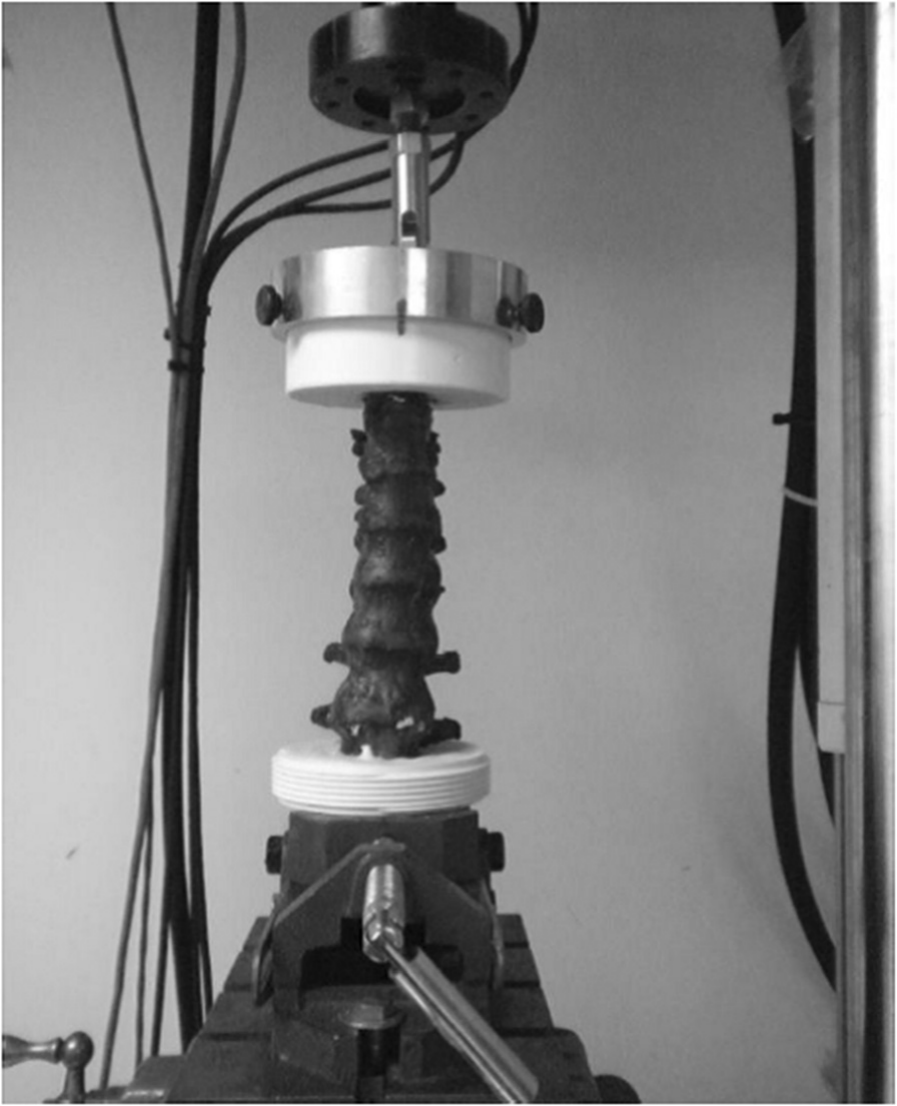
Photograph showing a reconstructed thoracolumbar specimen (T8-L2) positioned and clamped in the materials testing machine. The specimen is shown, as it would be, when loaded in pure axial compression

**Fig. 2:**
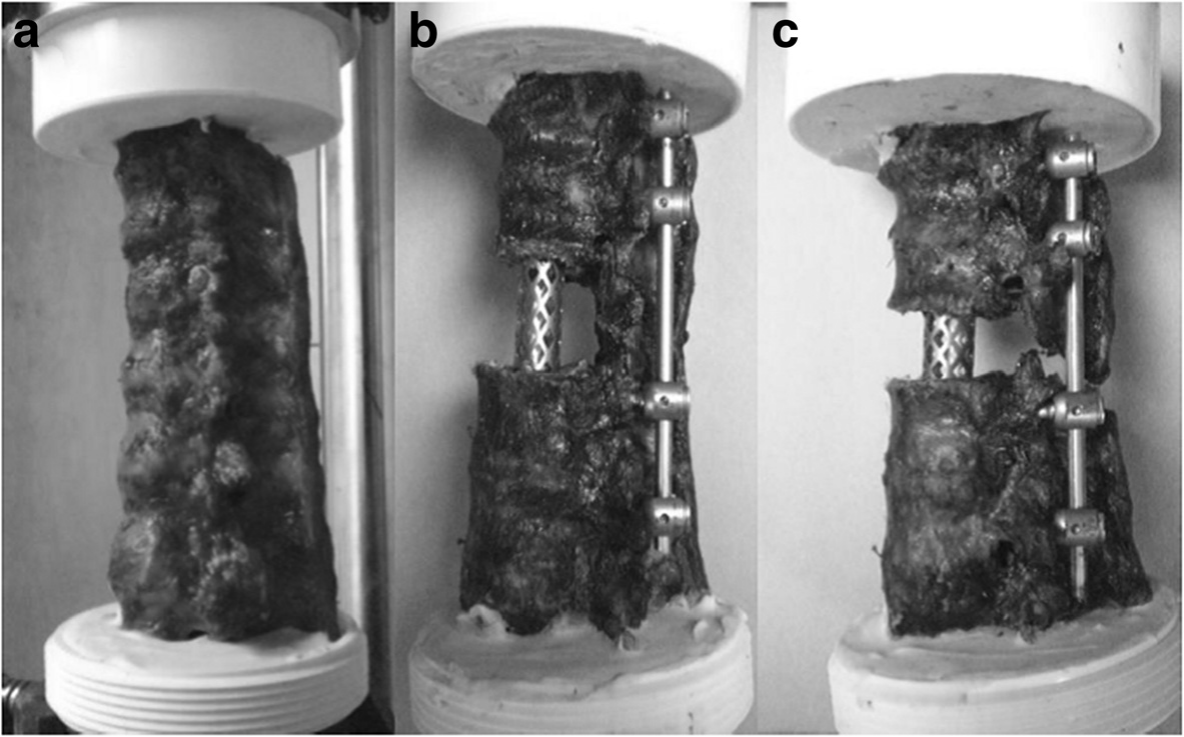
An intact specimen and the two reconstruction models. A Harms titanium mesh cage (17 × 22 mm in the cross section; Depuy Spine) was used as an anterior spacer in all reconstruction models. The posterior instrumentation was the Expedium pedicle screw systems (Depuy Spine): **a** intact, **b** anterior cage and multilevel posterior instrumentation at T9-L1, and **c** anterior 10-mm short cage and multilevel posterior instrumentation at T9-L1

**Fig. 3:**
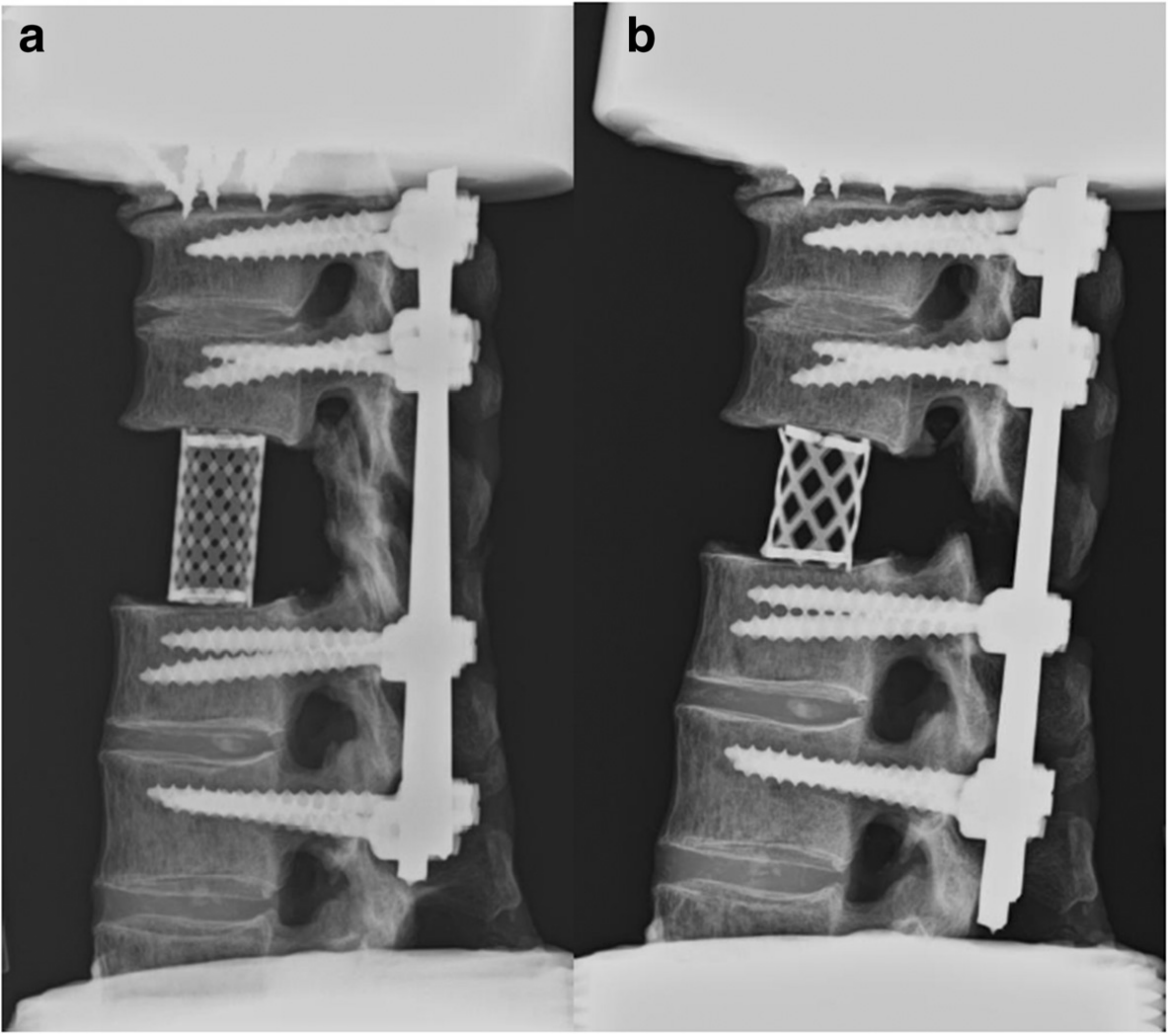
Lateral radiographs of the two reconstruction models. **a** After corpectomy, reconstruction with anterior cage and posterior instrumentation. **b** After total spondylectomy, reconstruction with anterior 10-mm shorter cage and posterior instrumentation

The biomechanical testing was carried out using a materials testing machine (MTS 858 Mini-Bionix Test System, Minneapolis, MN, USA). The vertebral bodies of T8 and L2 were potted in 10-cm diameter polyvinyl chloride endcaps using dental cement (Heraeus Kulzer Inc., South Bend, IN, USA). The specimen (T8-L2) in its two pots was then clamped on the testing machine in the upright position (Fig. 1). Each specimen was biomechanically tested three times, according to the following loading sequence (Figs. 2 and 3). Each specimen was tested intact, after corpectomy, and after total spondylectomy.

#### The intact specimen

Step 1 (establishing the center of rotation): The center of rotation for flexion-extension and lateral bending was established as follows. A pure compressive load of 50 N was applied through a roller bearing to the top surface of the upper end-cap containing T8. With the roller bearing positioned, the load was applied, then repositioned and applied again, until no angular rotation in the coronal or sagittal planes could be detected when a compressive force was applied to the upper end-cap. This spot on the top surface of the end-cap was deemed to be the center of rotation and remained as the reference spot in subsequent testing on that specific specimen. A cyclic compression force was then applied as conditioning (40 ± 10 N at 0.5 Hz for 15 min) to remove excess fluid from the discs and to return the disc to its predeath height [11].

Step 2 (compression): The specimen was then loaded in pure compression (with the load applied at the center of rotation) at a displacement rate of 0.25 cm/min. The load was applied up to a maximum of 200 N. A load-deformation curve was obtained. The specimen was then loaded two more times, and load-deformation curves were obtained each time. The three load-deformation curves were essentially identical, and only one was used to calculate the stiffness. Stiffness was calculated as the slope of the linear portion of the load-deformation curve [11].

Step 3 (flexion): The test was repeated with a 100 N load applied 2 cm anterior to the center of rotation, to produce a maximum bending moment of 2 Nm (i.e., 0.02 m × 100 N = 2 Nm) in flexion. The load was then released and applied twice more. The three load-deformation curves were essentially identical, and only one was used to calculate the stiffness.

Step 4 (extension): The test was repeated with a 100 N load applied 2 cm posterior to the center of rotation, to send the specimen into extension. The load was then released and applied twice more. The three load-deformation curves were essentially identical, and only one was used to calculate the stiffness.

Step 5 and 6 (lateral bending): The test was repeated with a 100 N load applied 2 cm to the right of the center of rotation, to send the specimen into right lateral bending. The load was then released and applied twice more. Three load-deformation curves were obtained for right lateral bending. The three right load-deformation curves were essentially identical, and only one was used to calculate the stiffness. The experiment was then repeated for left lateral bending, and the same criteria applied.

Step 7 and 8 (right and left axial rotation): To apply axial rotation, the specimen was first compressed to 100 N, and an axial torque was then applied in a clockwise motion (about the center of rotation) to a maximum of 10°. Three torque-angle deformation curves were obtained for right rotation. The three right torque-angle deformation curves were essentially identical, and only one was used to calculate the stiffness. The experiment was then repeated for left rotation, and the same criteria applied.

#### The corpectomy model

After the test on the intact specimen had been carried out (as described above), pedicle screws were inserted at T9, T10, T12, and L1 (5.5-mm diameter pedicle screws, 40-mm long at T9 and T10; 6.5-mm diameter pedicle screws, 45-mm long at T12 and L1 (Expedium System, Depuy Spine., Raynham, MA, USA)). A corpectomy at T11 was performed by incising and removing the T10-11 and T11-12 intervertebral discs and the cartilaginous endplates. The entire T11 vertebral body was removed after creating bilateral osteotomies through the T11 pedicles and resecting the anterior and posterior longitudinal ligaments at T11; the posterior elements were left intact. Vertebral body reconstruction was performed by implanting a Harms titanium mesh cage (Harms Cage, Depuy Spine) between T10 and T12, depending on the corpectomy height (Figs. 2 and 3). The diameter of each cage was 16 mm. After the corpectomy reconstruction had been assembled, the biomechanical tests (step 2 through step 8 as described above) were carried out on the specimen, and stiffness curves were obtained, as described above.

#### The total spondylectomy model

After the test on the corpectomy model had been carried out, the rod on one side was removed to allow access for the removal of the posterior elements. A total spondylectomy at T11 was carried out by removing the posterior elements. A cage 10 mm shorter than the one used in the corpectomy model was inserted at T11. After the “short” cage was inserted into the anterior defect, the posterior instrumentation was adjusted, using a compressor and rod-screw junctions, to compress the inserted cage (Figs. 2 and 3). With the total spondylectomy model assembled, the biomechanical tests (step 2 through step 8 as described above) were then carried out on the specimen, and stiffness curves were obtained, as described above.

Thus, each specimen was biomechanically tested three times: intact, as a corpectomy model, and as a total spondylectomy model. The order of assembling and testing the models was the same for all five specimens.

### Statistical analysis

Results for stiffness are presented as the mean of determinations, with error bars representing standard deviations. Means and standard deviations were computed for the five specimens, for each measure under each experimental condition. A repeated measures analysis of variance with the three models (intact, corpectomy, and total spondylectomy) was performed, followed by post hoc pairwise comparisons based on a Bonferroni correction. Post hoc comparisons were made for the intact spine versus each of the two models and for the corpectomy model versus the total spondylectomy model. The level of significance was *P* < 0.05. For statistical analysis, SAS version 9.2 (Cary, NC, USA) was used.

## Results

None of the specimens fractured, and none of the pedicle screws or rods loosened during the experiments. Visual inspection revealed no major pedicle violations. There was one minor lateral perforation, with threads barely visible outside of the pedicle, but this did not compromise fixation.

**Fig. 4:**
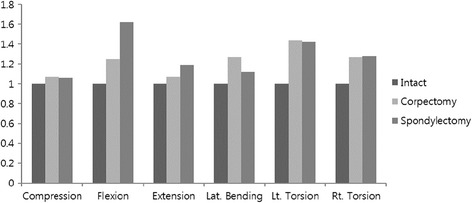
Means and standard deviations of the stiffness ratios compared to the intact specimens. No statistical significance was observed between the intact condition and each of the two reconstruction models or between the two reconstruction models

The mean ± standard deviation (SD) values of the stiffness values in axial compression, flexion, extension, lateral bending, and axial rotation are shown in Table 1 and Fig 4.

**Table 1:** Means and standard deviation of stiffness

Loading mode	Intact	Corpectomy	Spondylectomy	*P*
Axial compression (N/mm)	235.0 ± 63.3	243.6 ± 45.5	240. 1 ± 60.6	0.972
Flexion (N/mm)	149.4 ± 38.6	168.1 ± 53.6	219.9 ± 78.4	0.192
Extension (N/mm)	228.2 ± 85.4	230.5 ± 98.2	246.3 ± 66.7	0.935
Lateral bending (N/mm)	171.8 ± 51.3	204.8 ± 50.9	179.8 ± 26.9	0.494
Right rotation (Nmm/degree)	580 ± 270	730 ± 260	690 ± 180	0.610
Left rotation (Nmm/degree)	620 ± 310	710 ± 260	680 ± 200	0.870

Mean ± SD values are shown

Overall ANOVA *F*-test *P* value, *P* < 0.05

### Axial compression

No significant difference was observed between the two reconstruction models, or between the intact specimen and either of the two reconstruction models.

### Flexion

Both reconstruction models provided greater stiffness than that shown by the intact specimen, but the differences were not significant. The total spondylectomy model provided greater stiffness than the corpectomy model, but the difference was not significant.

### Extension

The two reconstruction models provided greater stiffness than the intact specimen, but the differences were not significant. The total spondylectomy model provided greater stiffness than the corpectomy model, but the difference was not significant.

### Lateral bending

The values of stiffness measured for right and left lateral bending were averaged for each specimen, since each of our specimens showed some slight scoliotic changes (three left scoliosis, two right scoliosis). Both of the reconstruction models provided greater stiffness than that shown by the intact specimen, but the differences were not significant. The corpectomy model provided greater stiffness than the total spondylectomy model, but the difference was not significant.

### Left axial rotation

The two reconstruction models provided greater stiffness than that shown by the intact specimen, but the differences were not significant. The corpectomy model provided greater stiffness than the total spondylectomy model, but the difference was not significant.

### Right axial rotation

The two reconstruction models provided greater stiffness than that shown by the intact specimen, but the differences were not significant. The corpectomy model provided greater stiffness than the total spondylectomy model, but the difference was not significant.

## Discussion

Spinal pathologic conditions, such as fractures, tumors, and degenerative diseases, lead to spinal instability, deformity, and neural element compression [12–14]. The question is, How should corpectomy be approached and stabilized (anteriorly, posteriorly, or combined anteroposteriorly)? This has been the subject of debate for some time. Classic corpectomy in a piecemeal fashion leaves the posterior column intact, providing up to 40 % of residual segmental stability [4–6]. Various authors assessed stiffness of several stabilizations using corpectomy and total spondylectomy models; these authors demonstrated that 360° stabilization had higher primary stiffness values, compared to posterior or anterior alone. A 360° stabilization is sometimes preferred, since it stabilizes a spinal segment on both sides of the center of rotation [4, 15–17]. More recently, a combination of anterior strut graft or cage and multilevel posterior instrumentation with pedicle screws by an anterior-posterior approach is commonly performed after corpectomy for the treatment of various spinal pathological conditions, such as fractures, tumors, and deformities. However, when transferring these results to clinical application, a combination of anterior strut graft and multilevel posterior instrumentation after corpectomy requires prolonged operative time and increases the surgical risks, including enlarged surgical approaches, increased cardiopulmonary complications, and greater blood loss [12, 18, 19]. Therefore, posterior total spondylectomy can be useful in cases where the anterior approach is considered difficult, such as in cases of severe adhesions due to previous anterior surgery, suboptimal pulmonary function, severe kyphosis, trunk shortening, and poor general health status [1–3].

Total spondylectomy (complete resection of the affected vertebra, including the posterior column) leads to complete loss of spinal continuity and stability. Therefore, subsequent secure and stable spinal reconstruction is absolutely required. Some authors reported that applied compression forces on the anterior strut graft or cage using posterior instrumentation improves stability [16, 20, 21]. As noted above, Kato carried out a biomechanical study using human cadavers and recommended reconstruction using a cage 10 mm shorter in order to provide more stability after total spondylectomy [7]. One reason for using a 10-mm shorter cage after total spondylectomy in our study is that in an actual spinal surgery, it can be very difficult to replace the removed vertebral body with a cage of the exact height. It is therefore common to use a shorter cage for the removed vertebral body. In addition, a compressive force is usually applied to the cage by means of posterior instrumentation to increase spinal stability in reconstruction. This maneuver results in slight spinal shortening (5–10 mm). A 10-mm shortening is within the safe range for the spinal cord, according to a report by Kawahara et al., who studied the safety limits and physiological effects of acute spinal shortening on the spinal cord in dogs and reported an increase in spinal cord blood flow, as well as no spinal cord injury [10].

In order to evaluate spinal stability after a total spondylectomy, the stiffness of the reconstructed spine and the stress generated in the instrument used for the spinal reconstruction have been investigated by a finite element method and loading test using a cadaveric spine. Experimentation using a cadaveric spine is a direct means to analyze the behavior of the implant through the displacement response of instrumented segments to applied forces, compared to intact segments. However, experimental results are sensitive to limitations caused by testing procedures and variability among specimens. A finite element model presents a great advantage, because it enables the same procedure (number of vertebrae in the segment, boundary conditions) and the same vertebral segment (geometry, mechanical characteristics) to be used for different spinal implants and for each implant to analyze the influence of a different parameter. Therefore, a validated finite element model would constitute a powerful simulation tool, for the clinician as well as for the implant designer [22]. Nevertheless, as far as we are aware, there is no published report on direct biomechanical comparison of 360° stabilizations for a corpectomy and a total spondylectomy in a finite element method or cadaveric models. This lack of clear-cut, relevant biomechanical data has meant that choosing an anterior corpectomy and a posterior total spondylectomy model has been based mainly on empirical evidence. Therefore, we compared biomechanical stabilities, using a thoracolumbar reconstructed spine (T9-L1) with anterior cage and multilevel posterior instrumentation after corpectomy (T11), and the same spine with a 10-mm shorter anterior cage and multilevel posterior instrumentation after total spondylectomy (T11).

Posterior pedicle screw segmental instrumentation could allow more rigid fixation with three columns. The length of the posterior fixation is a major determinant for rigidity of the construct. Long posterior fixations were significantly more stable compared to intact specimens. The functional relationship between length of posterior fixation and spinal stiffness following spondylectomy and subsequent reconstruction is emphasized by the decreased range of motion in the long posterior fixation group, when compared to short fixation and an intact specimen. In most of the biomechanical studies dealing with corpectomy or total spondylectomy models, an anterior cage combined with multilevel posterior fixation (two above and two below) was able to provide more stiffness than the intact specimens. On the other hand, there is evidence to suggest that eliminating mechanical loads on healing bone when using rigid fixation may result in negative bone remodeling and net bone loss. Thus, less rigid fixation that permits a certain degree of micromotion may accelerate the time to union. Akamaru et al. made a three-dimensional finite element model of the reconstructed spine (T10-L4) following total spondylectomy at T12. They concluded that a reconstruction method with multilevel posterior instrumentation (two above and two below) and no anterior fixation should be better at allowing stress for remodeling of the bone graft inside the titanium mesh cage [23]. Therefore, we concluded that four-level pedicle screw instrumentation (two above and two below) is an adequate method of reconstruction after spondylectomy.

There was no significant difference in stiffness after reconstruction of total spondylectomy versus corpectomy in this cadaveric model. Additionally, there was no significant difference in stiffness between the intact specimen and either of the two reconstruction models. Despite the additional bone resection performed when doing a total spondylectomy versus corpectomy, the constructs did not differ biomechanically. Some authors similarly refer to the importance of applied compression forces on the anterior strut graft to improve stability [16, 20, 21]. The stabilizing effect of such compression in reconstruction using a 10-mm shorter cage after total spondylectomy is supported by the results of our tests. This study made a direct biomechanical comparison between anterior cage and multilevel posterior instrumentation after corpectomy and anterior shorter cage (10 mm) and multilevel posterior instrumentation after total spondylectomy. We would expect that total spondylectomy using a 10-mm shorter cage would restore stiffness to a level equivalent to or greater than that of the intact spine and anterior corpectomy using a cage. In general, postoperative stability after reconstruction is accomplished by spinal fusion. Spinal internal fixation devices provide stability until spinal fusion occurs. Therefore, our own results show that each of the two reconstruction models restored stiffness to a level equivalent to that of spinal fusion in actual spinal surgery. In other words, posterior total spondylectomy using a 10-mm shorter cage can provide sufficient stability.

Several limitations to the study should be considered. 1) The loads and torques chosen to test the specimens in various configurations were low. We chose low loads and torques since there were many testing steps, and we wanted to avoid damage to the specimens before the entire sequence had been completed. 2) Our three models were tested immediately after reconstruction, so conclusions cannot be drawn about the long-term stability in a spine subject to cyclic loads applied daily. 3) The relatively small sample size may also be a limitation, although the results are very clear (no significant differences). The vagaries of postmortem collection always present problems. Most of the spines available for collection and subsequent testing are usually from older cadavers and often have multiple massive osteophytes that render the spines unsuitable for the testing described above. Most of the thoracic spines made available to us did not meet our selection criteria. 4) Thoracolumbar specimens were used in the present study, and only a T11 spondylectomy model was evaluated. Corpectomy and total spondylectomy are performed not only in the lumbar spine but also in the proximal and midthoracic spine. The biomechanical environment of the thoracic spine is quite different from that of the lumbar spine because of the stabilizing effects of the costovertebral joints and rib cage. T11 at the thoracolumbar junction is exposed to forces similar to the lumbar spine. Therefore, reconstruction stability after total spondylectomy in the thoracic spine remains unknown. Further biomechanical studies examining the comparative multisegmental mechanics of total spondylectomy in the thoracic region are required for certainty. 5) The results of the present study do not allow for a recommendation regarding the length of constructs to be used clinically, when performing either total spondylectomy or corpectomy. Depending on the bone purchase achieved by the pedicle screws, as well as the overall sagittal alignment, additional pedicle screw fixation may be needed in clinical practice.

The choice of management for spinal surgery depends on many factors, which surgeons must weigh before determining the ideal surgical approach. These in vitro biomechanical data provide helpful guidelines for planning surgical management.

## Conclusion

A classic corpectomy, which leaves the posterior column intact, is no better in terms of stability/stiffness than a total spondylectomy, when the total spondylectomy is carried out using a shorter cage followed by compression using posterior instrumentation.
